# Assessing peer review by gauging the fate of rejected manuscripts: the case of the Journal of Artificial Societies and Social Simulation

**DOI:** 10.1007/s11192-017-2241-1

**Published:** 2017-03-03

**Authors:** Niccolò Casnici, Francisco Grimaldo, Nigel Gilbert, Pierpaolo Dondio, Flaminio Squazzoni

**Affiliations:** 1 Department of Experimental and Clinical Sciences, University of Brescia, Brescia, Italy; 2 Departament d’Informàtica, University of Valencia, Avinguda de la Universitat, Burjassot, València Spain; 3 Centre for Research in Social Simulation, Department of Sociology, Faculty of Arts and Human Sciences, University of Surrey, Guilford, UK; 4 School of Computing, Dublin Institute of Technology, Dublin, Ireland; 5 Department of Economics and Management, University of Brescia, Via San Faustino 74/b, 25122 Brescia, Italy

**Keywords:** JASSS, Peer review, Rejected manuscripts, Impact factor, Citations

## Abstract

This paper investigates the fate of manuscripts that were rejected from *JASSS*-*The Journal of Artificial Societies and Social Simulation*, the flagship journal of social simulation. We tracked 456 manuscripts that were rejected from 1997 to 2011 and traced their subsequent publication as journal articles, conference papers or working papers. We compared the impact factor of the publishing journal and the citations of those manuscripts that were eventually published against the yearly impact factor of *JASSS* and the number of citations achieved by the *JASSS* mean and top cited articles. Only 10% of the rejected manuscripts were eventually published in a journal that was indexed in the Web of Science, although most of the rejected manuscripts were published elsewhere. Being exposed to more than one round of reviews before rejection, having received a more detailed reviewer report and being subjected to higher inter-reviewer disagreement were all associated with the number of citations received when the manuscript was eventually published. This indicates that peer review could contribute to increasing the quality even of rejected manuscripts.

## Introduction

Looking at the fate of manuscripts previously rejected from a journal is important in evaluating the quality of peer review and a journal’s position and prestige in the publishing market (e.g., Bornmann 2011) for a number of reasons. First, considering the common features of rejected manuscripts can help to evaluate possible authors’ misperceptions about the journal’s scope and reveal coordination problems between authors and journals. This could include authors’ misunderstanding of the target of their work or journals communicating policy signals that confuse authors. Secondly, this analysis can prove the existence of different sources of editorial or reviewer bias (e.g., Siler et al. 2015; Weller 2002). Thirdly, while examining rejected manuscripts means focusing mainly on the selection side of peer review, tracing the trajectory of rejected submissions after the original peer review can show if peer review can also increase the quality of the rejected manuscripts for future publication (e.g., Walker and Rocha da Silva 2015).

Previous studies of the fate of rejected manuscripts looked, first, at the effect of rejections in delaying publication, by considering the relationship between the impact factor of the rejecting journal and that of the journal that eventually published the manuscript. This was to understand authors’ publication strategies, e.g., trying for publication in a highly ranked journal first and then successively in less prestigious ones, and to estimate potential editorial bias. Some studies have also tried to estimate whether peer review could help authors to increase the quality of their rejected submissions by understanding what authors modified when targeting subsequent journals.

For instance, in one of the pioneering works in the field, Chew (1991) examined the fate of 254 rejected manuscripts that were originally submitted in the first 5 months of 1986 to the *American Journal of Roentgenology*, an influential journal of diagnostic radiology. He reconstructed the trajectory of these manuscripts 45–54 months after the journal’s rejection. He found that the mean time lapse between rejection and later publication was 15 months and that most of the rejected manuscripts were published in journals with a lower impact. Ray, Berkwits, and Davidoff (2000) performed a similar study of 350 manuscripts that were rejected by the *Annals of Internal Medicine* in 1993–1994. 69% of them were eventually published, mostly in specialty journals of lower impact after a mean of 18 months. They also found that time to publication had a weak negative correlation with the impact factor of the journal in which the article was published (correlation coefficient −0.15, *p* = 0.007). Similar results were found by Opthof et al. (2000) in a study of 644 manuscripts that were rejected by *Cardiovascular Research* in 1995–1996, by Nemery (2001) in a study of *Occupational and Environmental Medicine* in 1995–1997 and by Liesegang, Shaikh, and Crook (2007) in a study of the *American Journal of Ophthalmology* in 2002–2003. Investigating 366 manuscripts that were rejected by the *Journal of Vascular and Interventional Radiology* in 2004, Silberzweig and Khorsandi (2008) were able to determine that by 2007 58% had been published in other journals and as a result of the initial rejection there had been a delay of 15.5 months in these manuscripts achieving publication.

McDonald, Cloft, and Kallmes (2007) examined a sample of manuscripts that were rejected by the *American Journal of Neuroradiology* during 2004. They considered submission type (i.e., major study, technical note or case report), publication delay, publishing journal type (i.e., neuroradiology, general radiology, or clinical neuroscience journal) and impact factor. They found that of the 554 rejected submissions, 315 (56%) were subsequently published in 115 different journals, with the *Journal of Neuroradiology* publishing the greatest number of articles (37 [12%]). The mean publication delay was 15.8 ± 7.5 months. The mean impact factor of journals subsequently publishing rejected manuscripts was 1.8 ± 1.3, compared with the impact factor of the *American Journal of Neuroradiology*, which was 2.5. 24 (7.5%) manuscripts were subsequently published in journals with higher impact factors than the rejecting journal. A later study by the same authors using Scopus data (McDonald et al. 2009) about articles published in the *American Journal of Roentgenology* showed that rejected articles that subsequently found a publication outlet had a lower number of citations than the average article in the rejecting journal, and that the number of citations received was correlated with the impact factor of the publishing journals. The numbers of citations received were higher in case of technical reports and when the publishing journals were close in subject matter to the *American Journal of Roentgenology*.

Wijnhoven and Dejong (2010) examined 926 manuscripts rejected by the *British Journal of Surgery* and found that 609 (65.8%) were published in 198 different journals, mostly in subspecialty surgical and non-surgical journals with a mean time lapse of 13.8 months. Only 14 manuscripts (2.3%) were eventually published in journals with a higher impact factor than the *British Journal of Surgery*. Similar results were found by Khosla et al. (2011) in a study on 371 manuscripts that were rejected by *Radiology* in 2005–2006, although here the mean delay was 17.3 months. Similar results were obtained in a retrospective online survey by Hall and Wilcox (2007) on a sample of authors rejected by *Epidemiology* in 2002. In general, authors admitted that their manuscripts that were rejected by the first journal were ultimately submitted to a journal of lower impact, so confirming the hypothesis that authors first try prestigious journals and then go for less prestigious ones.

An example of the analysis of potential editorial bias is Vinther and Rosenberg (2011), which found that publication of previously rejected submissions could be less likely if the previously rejecting journal was a non-English-language journal while the subsequent target was an English-language outlet. More recently, Holliday et al. (2015) looked at 500 manuscripts submitted to the *International Journal of Radiation Oncology*-*Biology*-*Physics* in 2010–2012 and tried to estimate whether rejected manuscripts could have been penalized by bias due to gender, country, academic status and the prestige of the submitting authors. While they found that there was no significant difference in acceptance rates according to gender or the academic status of the submitting authors, there were significant differences due to the submitting author’s country and h-index. By July 2014, 71.7% of the rejected manuscripts had been published in a PubMed-listed journal. Confirming previous results, the publishing journals had a lower impact factor and the published version of the manuscripts a lower number of citations compared to those published by the rejecting journal.

More interestingly, especially to understand whether peer review contributes to increasing the quality of rejected manuscripts for future publication, Armstrong et al. (2008) examined the case of 489 manuscripts rejected by the *Journal of the American Academy of Dermatology* in 2004–2005. They looked at whether the authors of rejected manuscripts adopted in their final publications the changes suggested by the original journal reviewers. Among the 101 subsequently published manuscripts for which full texts were available, 82% of the authors incorporated at least one change suggested by the original reviewers. These manuscripts were eventually published in journals with higher impact factors than those that did not incorporate any reviewer suggestions (*p* = 0.0305). A more in depth-study on *Angewandte Chemie International Edition* by Bornmann, Weymuth and Daniel (2010), who applied a content analysis to referee reports on 1899 manuscripts that were reviewed in 2010, confirmed a relation between original peer review and later publication of rejected manuscripts. While 94% of the 1021 rejected manuscripts were published more or less unchanged in another journal, they found that previously rejected manuscripts were more likely to be published in journals of higher impact factor when there were no negative comments by reviewers on important aspects of the submission, such as relevance of contribution and research design.

However, given that evaluation and publication time delays are strongly field dependent and that the publishing market is highly stratified and segmented from field to field, these studies may only be relevant to the practices and informal norms of research in medicine and related fields. It is not clear whether the findings are context-specific or can inform us about trends that are more general. Furthermore, these studies were constrained by a limited time frame, typically following papers for only a couple of years after they were rejected. This may be sufficient in fields such as medicine, but not for others, e.g., computer science, social sciences and humanities, where there are more types of publication outlet, including conference proceedings and books, and longer publication trajectories (e.g., Powell 2016).

To fill this gap, we reconstructed the fate of unpublished manuscripts in a multidisciplinary journal, the *Journal of Artificial Societies and Social Simulation* (from now on, *JASSS*), an open access, online interdisciplinary journal for the exploration and understanding of social processes by means of computer simulation. We examined 14 years of submissions so as to look at the longer publication trajectories that are more typical in social science. We analysed the type of publications that eventually resulted from rejected manuscripts and the journals in which later versions of some of these manuscripts were published. Then, we measured the impact factor of the journal that published each rejected manuscript and counted the citations that the article eventually received. We used this to estimate whether, when it rejected manuscripts, *JASSS* lost important contributions.

The rest of the paper is structured as follows. "Data" section presents our dataset, including data from the journal management system and data we extracted from other sources. "Results" section presents the results. "Discussion and conclusions" section discusses the main limitations of our study and suggests measures to make this type of analysis easier.

## Data

Established in 1998 and indexed in ISI, Scopus and other major journal databases, *JASSS* (see: http://jasss.soc.surrey.ac.uk/) is the flagship journal of social simulation, i.e., the study of social processes through computer simulation. The journal received 1272 submissions and published 606 articles and 236 book reviews during the period from 1st January 1998 to 24th February 2015. The proportion of article submissions rejected by the editor increased from 50% in 2006 to 75% in 2015. The journal applies a double blind model of peer review, has an average decision time of approximately 60 days from author submission to the editorial decision and on average, referees report within 30 days. It is truly international with about 20% of first submission authors from the US, 13% from UK, 10% from Germany, 9% from China and the rest worldwide, from Japan to Australia (source: internal journal statistics).

The data analysed covered 14 years of submissions to *JASSS*, from 1997 to 2011. Data were extracted on 12 May 2012 from the epress system used by the journal to manage submissions and reviewing (http://www.epress.ac.uk), with the agreement of the journal editor at the time. Data included 1008 submissions, of which 456 were rejected manuscripts. For each submission, we had submission date, referee recommendations, review rounds and editorial decision. We also considered the status of the first author (classified as senior, i.e., associate or full professor, or junior, i.e., PhD student or post-doc) and the length of the review reports, because these factors were found to have an important role in a previous study on the quality of peer review in *JASSS* (Casnici et al. 2016).

In order to trace the status of the rejected manuscripts, we first searched manually on Google Scholar for any record that had the same title as the rejected submission and included the corresponding author of the original submission among its authors. This gave us a first list. Then, we searched any record that shared the same words of the submission title and included the corresponding author of the original submissions among its authors. In this case, we restricted the search to any word composed of more than seven letters as we assumed that these words were more informative and would have been retained even if there had been some change in the wording of the title. This gave us additional records, whose correspondence with rejected submissions was checked manually case-by-case. These two steps gave us 131 manuscripts. Finally, we searched on the Web using Google Search Engine for any record that had words in the title that were similar to the rejected submission titles. This gave us 52 further records, whose correspondence with rejected submissions was checked manually case-by-case.

We used the Web of Science (WoS) to extract the impact factors of the journals in which rejected manuscripts were eventually published and Google Scholar to extract citation data about the published articles, as well as for the articles originally published in *JASSS*. In order to compare the number of citations of published versions of rejected manuscripts with the number of citations of articles published in *JASSS*, we calculated the number of citations received by the average and top cited *JASSS* articles year by year. Then, we compared the number of citations received by each article rejected by *JASSS* and published elsewhere with the average number of citations received by articles published in *JASSS* in the same year and by the top 10 cited *JASSS* articles in that year. We did the same when we compared the impact factor of *JASSS* and that of other journals publishing previously rejected manuscripts.

## Results

We were able to trace the trajectory of 183 (40.1%) of the 456 manuscripts rejected by *JASSS*. The remaining 273 were probably never published, published in non-indexed sources or changed so dramatically as to make linkage back to the original submission impossible to trace. Of these 183 rejected manuscripts, 88 (48.1%) were eventually published in a journal (see Table 1), of which 46 (25.1%) were published in journals that were indexed in WoS (Table 2). A further 24 (13.1%) were published in conference proceedings (Table 3).

**Table 1:** Destination of the manuscripts rejected from *JASSS* (*Source*: Google Scholar)

Type of publication	Freq.	Percent
Book	1	0.5
Book chapter	8	44
Conference proceedings	24	13.1
Journal article	88	48.10
Working paper	62	33.9
Total	183	100.00

**Table 2:** The journals in which manuscripts rejected from *JASSS* were eventually published (*Source*: WoS and Google Scholar)

Journal	Number of published articles
**Complex Systems (6.8%)**	**6**
Complex Systems	1
Complexity*	2
Connection Science	1
Fluctuation and Noise Letters*	1
Interdisciplinary Description of Complex Systems	1
**Computer science (51.1%)**	**45**
International Journal of Information Technologies and Systems Approach	1
Advances in Computer Science and Engineering	1
Advances in Systems Science and Application	1
AI and society	1
Applied Artificial Intelligence*	1
Artificial life*	1
Electronic International Journal of Time Use Research	1
Expert Systems with Applications*	3
Information and Knowledge Management	1
Information Sciences	1
International Journal of Advancements in Computing Technology	1
International Journal of Computer Science and Information Technology	1
International Journal of Knowledge and Systems Science (IJKSS)	1
International Journal of Knowledge-based and Intelligent Engineering Systems	1
International Journal of Microsimulation	2
International Journal of Modelling and Simulation	1
International Journal of Social Network Mining	1
International Journal of System Dynamics Applications (IJSDA)	1
Journal of Information and Optimization Sciences	1
Journal of Modelling and Simulation of Systems	1
Journal of System Simulation	1
Journal of the American Society for Information Science and Technology*	1
Journal of the Brazilian Computer Society	1
Journal of the Operational Research Society*	1
Journal of the Operations Research Society of Japan*	1
Knowledge based systems*	1
Lecture Notes in Computer Science*	2
Optics and Laser Technology*	1
OR insight	1
Revista Brasileira de Computação Aplicada	1
Scientia Iranica*	1
SICE Journal of Control, Measurement, and System Integration	1
Simulation*	2
Socially Inspired Computing	1
Special Issue: Advances in Computer Science and Engineering	1
Studies in Informatics and Control	1
The International Journal of Virtual Reality	1
Transaction of the Japan Society for Simulation Technology	1
Transportation planning and technology*	1
Web Intelligence and Agent Systems: An International Journal	1
**Economics, management and business (14.8%)**	**13**
American Journal of Economics*	1
Developments in Business Simulation and Experiential Learning	1
International Journal of Production Research*	2
International review of financial analysis	1
Journal of Business Ethics*	1
Journal of Economic Interaction and Coordination*	2
Journal of Engineering Business Management	1
Journal of Game Theory	1
JTAER	1
Middle Eastern Finance and Economics	1
Studies in Nonlinear Dynamics and Econometrics*	1
**Geography (5.7%)**	**5**
Annals of the Association of American Geographers*	1
Computers, Environment and Urban Systems	1
Cybergeo: European Journal of Geography	1
International Journal of Geographical Information Science*	1
Journal of geographical systems*	1
**Medicine (5.7%)**	**5**
Computational and Mathematical Methods in Medicine*	2
Computers in biology and medicine*	1
Interactive Cardio Vascular and Thoracic Surgery*	1
International Journal of Systematic and Evolutionary Microbiology*	1
**Social science (5.7%)**	**5**
Computational and Mathematical Organization Theory*	1
Social Science Computer Review*	2
Social science information*	2
**Others (10.2%)**	**9**
Mind and Society	1
Ecological Economics*	1
Acta Biotheoretica*	2
Agricultural systems*	1
Journal of the Chemical Society of Pakistan*	1
PLoS ONE*	1
Trakya Univ J Sci	1
International Journal of Modern Physics C*	1

* Indicates journals that were indexed in WoS when the article was published. *Lecture Notes in Artificial Intelligence* is included because it is a well-established book series fully indexed in WoS and Scopus and it uses an evaluation procedure that makes it closer to a journal than to a book or conference proceedings

**Table 3:** Conference proceedings in which manuscripts rejected from *JASSS* were eventually published (*Source*: WoS and Google Scholar)

Conference proceedings	Number of published papers
International Workshop on IEEE	1
2013 46th Hawaii International Conference on System Sciences	1
8th World Multiconference on Systemics, Cybernetics and Informatics (SCI 2004)	1
AAAI Spring Symposium: Technosocial Predictive Analytics	2
European Social Simulation Association	1
Hawaii International Conference on System Sciences	1
IASTED International Conference 2005	1
ICICTA International Conference	1
IEEE Congress on	1
International System Dynamics Conference	1
KIIE/KMS Conference	1
Proceedings of the 11th IEEE International Symposium on Distributed Simulation and Real Time Applications. IEEE Computer Society	1
Proceedings of the 2003 Winter Simulation Conference	1
Proceedings of the 2010 3rd International Conference on Emerging Trends in Engineering and Technology	1
Proceedings of the 37th Hawaii International Conference on System Sciences	1
Proceedings of the 5th WSEAS international conference on Non linear analysis, non linear systems and chaos	1
Proceedings of the AISB 2009 Convention	1
SPIE Fourth International Symposium on Fluctuations and Noise	1
The 30th International Conference of the System Dynamics Society	1
The Geneva Papers on Risk and Insurance Issues and Practice	1
Third International Model to Model Workshop	2
Transportation, Mechanical, and Electrical Engineering (TMEE), 2011 International Conference	1

One might think that authors whose manuscripts had been rejected by *JASSS* would address their submissions to journals publishing in the same or closely related fields. A previous study (Squazzoni and Casnici 2013) mapped the scientific community surrounding *JASSS* and identified *Computational and Mathematical Organization Theory*, *Advances in Complex Systems* and *Lecture Notes in Artificial Intelligence* as some of the most similar journals to *JASSS* in terms of the subject matter of the papers they publish. However, these journals were not favoured by authors seeking a home for their rejected manuscripts; on the contrary, authors chose a remarkably wide variety of journals in which to publish (see Table 2).

### Impact factor of journals of eventual publication

**Fig. 1:**
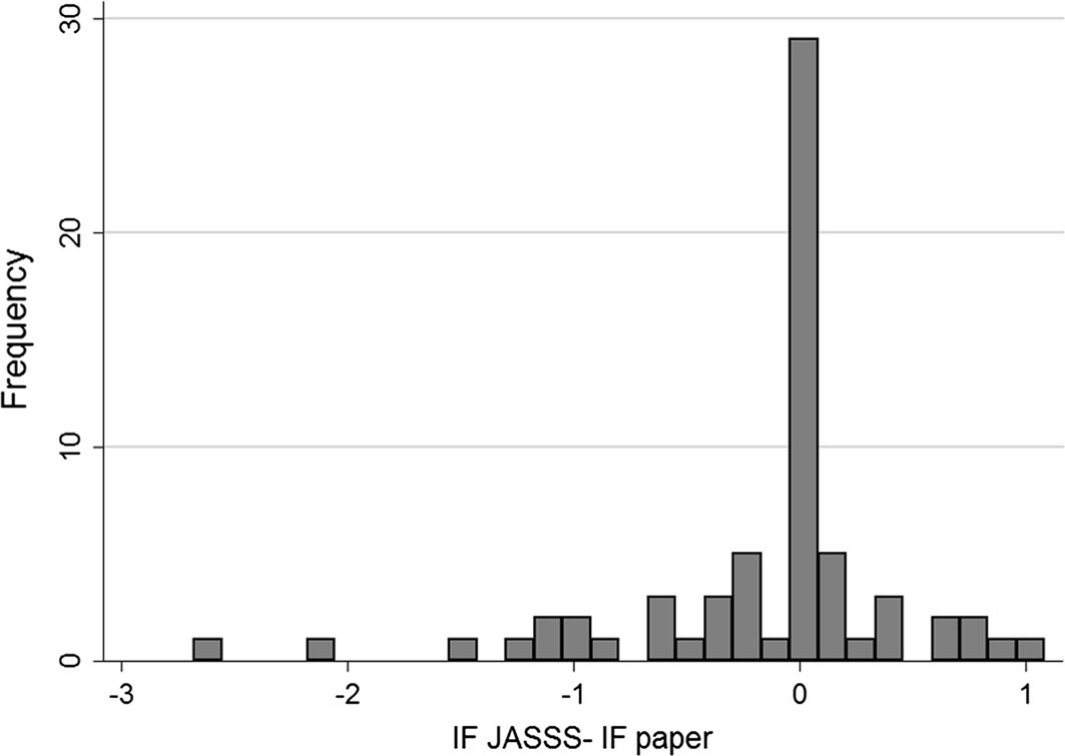
Impact factor of *JASSS* and other journals publishing previously rejected manuscripts (*source*: WoS). The *vertical axis* shows the number of published manuscripts, while the *horizontal axis* shows the difference between the impact factor of *JASSS* and that of the journal that published the manuscripts. Note that 0 means that the impact factor of *JASSS* and that of other publishing journals in the year when the manuscripts were eventually published was the same. Negative values arise from cases for which the impact factor of journals in which previously rejected articles were published was higher than *JASSS* in the same time window

Figure 1 shows the relationship between the impact factor of *JASSS* and that of the other journals publishing previously rejected manuscripts (the impact factors are those at the date when the manuscripts were eventually published, and are only for journals included in WoS).

43% of the manuscripts that were rejected from *JASSS* were later published in a journal with the same or lower impact factor compared to what *JASSS* achieved in the same year in which the manuscripts were published. Most rejected manuscripts were published in journals with only a slightly higher or lower impact factor. We found only a few exceptions, e.g., a manuscript previously rejected from *JASSS* that was published in *PLoS ONE*, which had more than twice the impact factor of *JASSS* (4.1 against 1.7) at the time the manuscript was published.

### Citations received by rejected manuscripts

Table 4 shows the mean number of citations that were received by manuscripts that were rejected from *JASSS* and later published in an indexed journal, an indexed conference proceedings or an indexed book. A comparison of these figures with the mean number of citations received by articles published in *JASSS*, which was 34.4 (standard deviation = 2.97), shows that by rejecting those manuscripts, *JASSS* generally did not reject contributions that were ultimately highly cited.

**Table 4:** Mean number of citations of published versions of the manuscripts rejected by *JASSS* (*Source*: Google Scholar)

Type of publication	Mean	SD
Book	2	–
Book chapter	3.86	2.79
Conference proceeding	4.27	6.58
Journal	13.74	28.49
Working paper	8.66	21.33
*JASSS*	34.4	2.97

**Fig. 2:**
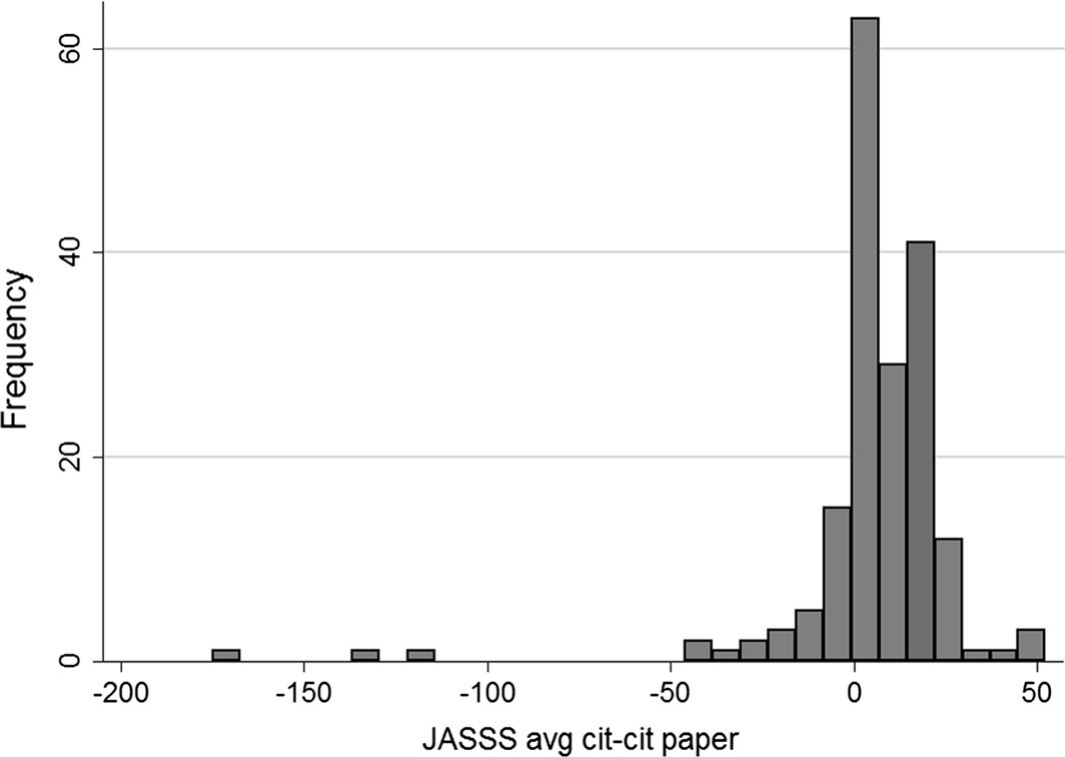
Differences between average citations of articles published in *JASSS* and citations received by manuscripts later published in other journals. The *vertical axis* shows the number of published manuscripts while the *horizontal axis* shows the difference between average citations of articles published in *JASSS* and citations received by manuscripts later published in other journals. Note that 0 means that average citations received by articles published in *JASSS* and citations received by previously rejected manuscripts were the same. Negative values means cases in which later published articles received disproportionaly more citations than articles published in *JASSS* in the same time window

57% of the previously rejected manuscripts were published in journals with an impact factor higher than *JASSS*, but only 38% of them received more citations than the articles published in *JASSS* in the same year (see Fig. 2).

We also calculated how many articles published elsewhere would have received sufficient citations to be included among the top 10 most cited *JASSS* articles. To do so, we measured citations received by the top 10 most cited *JASSS* articles in each year and compared them with the citations received by each manuscript that was eventually published in another journal in the same year. Only 6% of manuscripts previously rejected from *JASSS* and published elsewhere would have reached the top 10 (i.e., 11 of the 185 rejected manuscripts).

**Fig. 3:**
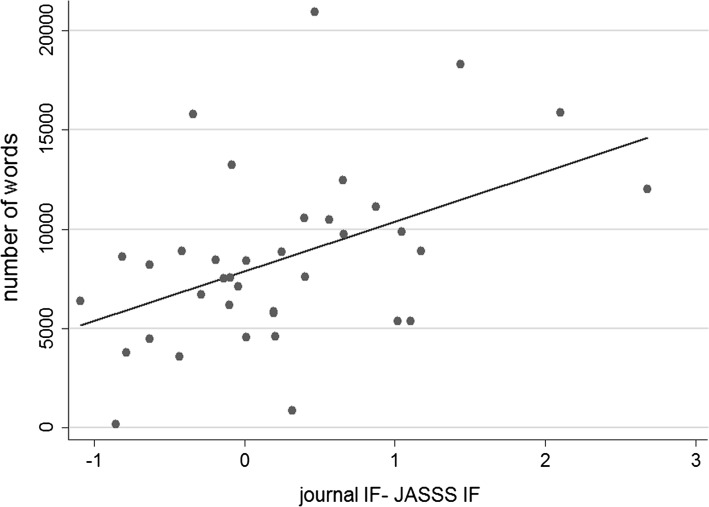
Relationship between the impact factor of journals that eventually published rejected manuscripts and the length of the reviewer reports that led to a rejection in *JASSS* (*source*: WoS). The* vertical axis* shows the number of words included in the reviewer reports, while the* horizontal axis* shows the difference between the impact factor of *JASSS* when manuscripts were rejected and the impact factor of the journals that published the *JASSS* rejected manuscripts at the time of their publication

All articles published elsewhere receiving a higher number of citations than the mean for *JASSS* went through more rounds of reviews before they were rejected than those articles that received fewer citations than the mean (an average of 1.47 rounds of reviews compared with 1.18). Similarly to Casnici et al. (2016), we tested whether the length of the reviewers’ reports, which we considered as a proxy of the quality of informative content of the review, was associated with the impact factor of the journal in which rejected manuscripts were later published. The length was measured by summing the number of words of the report’s text, excluding all review guidelines included in the journal’s review format and possible pleasantries used by the referees when reporting via e-mail (in such cases, the text was copied and pasted manually from the e-mails by the journal editor in the epress management tool. We found that there was a positive correlation between the length of the reviewers’ reports that a rejected manuscript received and the difference between the impact factor of the journal in which it was eventually published and that of *JASSS* in the same year (Pearson correlation coefficient = 0.46, *p* = 0.004) (see Fig. 3). Thus, the informative content of the reports was associated with a higher impact factor for the journal that eventually published the rejected manuscript.

We also found a positive correlation, although weaker (*T* test, *p* = 0.083), between the extent of inter-reviewer disagreement and the impact factor of the journal in which the manuscript was published. Similarly to Casnici et al. (2016), the level of inter-reviewer disagreement was measured by assigning a number to recommendations: accept (1), minor revision (2), major revision (3), and reject (4), and calculating the standard deviation of the reviewers’ recommendations. We assumed that the higher the standard deviation, the higher the level of disagreement between the reviewers. We found a positive correlation between the level of reviewer disagreement and the probability that the rejected manuscript, when published, received more citations than the mean number of citations of articles published in *JASSS* in the same year (*T* test, *p* = 0.044). This is in line with previous findings on the importance of peer review and reviewer disagreement in improving the quality of the manuscripts (e.g., Radicchi 2012). However, there was no significant correlation between the academic seniority of the first submission author (i.e., associate/full professor vs. PhD student/post-doc) and the number of citations received by the rejected manuscripts after they had been published, or the impact factor of the publishing journal., Neither the disciplinary background of the manuscript’s reviewers showed any correlation withthe number of citations received by the rejected manuscripts after they had been published, or the impact factor of the publishing journal.

## Discussion and conclusions

In our study, we were able to gauge the trajectory of about 40% of manuscripts rejected by *JASSS*. About half of these were eventually published in journals, but only half of those journals were indexed in WoS. Most of the remainder that could be traced were eventually published as conference proceedings or working papers. Although 57% of the rejected manuscripts were published in journals with an impact factor higher than *JASSS*, they received less citations on average than articles that were published in *JASSS* in the same time window.

These findings indicate that *JASSS* did not lose many potentially influential articles when rejecting submissions. Furthermore, manuscripts that were exposed to more intensive peer review in *JASSS* before being rejected generally had more success when they were eventually published. Although some analysts describe peer review as an unfair and unproductive mechanism for quality control (e.g., Clair 2015), in this case we found evidence that the review process avoided false negatives and had the effect of increasing the quality of even the unpublished manuscripts (Armstrong et al. 2008; Bornmann et al. 2010).

Defining the quality of peer review in scholarly journals only by gauging the fate of rejected manuscript is a first step towards more comprehensive measures. Measuring quality through journal impact factors and/or article citations is problematic, due to the disproportionate effect of a few highly cited articles (e.g., Baum 2011). Previous research also suggests that the quality of peer review is a complex concept, which strongly depends on the multiple, often ambiguous functions that this process has (e.g., Cowley 2015; Lamont et al. 2009; Ma et al. 2013; Pontille and Torny 2015; Ragone et al. 2013). For instance, current research on peer review sees it as an engine to select excellent, innovative or rigorous research and avoid publishing below standard contributions (Nedić and Dekanski 2016). Ideally, such a filter should make the outcome of the process objective, unbiased and predictable. An alternative perspective is to consider peer review as a form of cooperation between experts who are called to increase the value of scientific contributions (e.g., Bornmann et al. 2012). Gauging the fate of rejected manuscripts, as we have done here for a specific case, is key to understanding whether confidential peer review can work effectively not only as a filter, but also as a means to improve the quality of research.

Finally, as this study has shown, analysing the fate of rejected manuscripts is difficult. This is especially the case for disciplines such as the social sciences and humanities where a variety of publication outlets exist and there is a considerable delay between submission and publications (Verleysen and Engels 2014). Nevertheless, although many obstacles remain (e.g., Jefferson et al. 2002), modern online archives, data mining techniques and generally a higher sensitivity towards openness, data sharing and transparency among science stakeholders, including publishers, is likely to make this type of analysis easier in the near future. The availability of more accessible and better data would also help publishers and journal editors to understand the situation of their journals better and to establish more systematic quality controls.
